# Therapeutic use of stem cells for cardiovascular disease

**DOI:** 10.1186/s40169-016-0116-3

**Published:** 2016-08-18

**Authors:** Whitney Faiella, Rony Atoui

**Affiliations:** Division of Cardiac Surgery, Health Sciences North, 41 Ramsey Lake Road, Sudbury, ON P3E 5J1 Canada

**Keywords:** Myocardial infarction, Induced pluripotent stem cells, Mesenchymal stem cells, Cardiac stem cells, Stem cell therapy, Bone marrow, Adipose tissue

## Abstract

Stem cell treatments are a desirable therapeutic option to regenerate myocardium and improve cardiac function after myocardial infarction. Several different types of cells have been explored, each with their own benefits and limitations. Induced pluripotent stem cells possess an embryonic-like state and therefore have a high proliferative capacity, but they also pose a risk of teratoma formation. Mesenchymal stem cells have been investigated from both bone marrow and adipose tissue. Their immunomodulatory characteristics may permit the use of allogeneic cells as universal donor cells in the future. Lastly, studies have consistently shown that cardiac stem cells are better able to express markers of cardiogenesis compared to other cell types, as well improve cardiac function. The ideal source of stem cells depends on multiple factors such as the ease of extraction/isolation, effectiveness of engraftment, ability to differentiate into cardiac lineages and effect on cardiac function. Although multiple studies highlight the benefits and limitations of each cell type and reinforce the successful potential use of these cells to regenerate damaged myocardium, more studies are needed to directly compare cells from various sources. It is interesting to note that research using stem cell therapies is also expanding to treat other cardiovascular diseases including non-ischemic cardiomyopathies.

## Introduction

Myocardial infarction (MI) is a leading cause of morbidity and mortality worldwide [1]. MI occurring from coronary artery disease can cause reversible or irreversible ischemic damage, depending on the reperfusion status afterwards. This ischemic damage results in the loss of cardiomyocytes due to apoptosis [2]. Following the formation of necrotic myocardium, a secondary inflammatory immune response occurs as myofibroblasts are recruited to the area resulting in scar formation and reduced ventricular function [3].

Current options to re-perfuse occluded arteries include medical, percutaneous coronary intervention and surgical strategies, which have significantly improved outcomes after MI [4]. However, these techniques do not reverse necrotic or ischemic myocardium. Research is now focusing on techniques to regenerate damaged myocardium to regain heart function, one of which is the use of stem cells.

Studies are currently investigating a number of different cell types including embryonic, induced pluripotent, mesenchymal and cardiac derived stem cells. There are many considerations to be taken into account when selecting an ideal cell type. For example, in order to improve heart function, cells must be able to differentiate into myocytes, vascular endothelial cells and smooth muscle cells or must act via paracrine mechanisms. Their extraction and isolation must be feasible and transplantation into humans must be safe and effective. In the following review, different cell types will be discussed in terms of the benefits and limitations of each type (Table 1).

**Table 1:** Summary of advantages, disadvantages and current clinical trials for the various sources of stem cells mentioned in this article

Cell type	Advantages	Disadvantages	Current clinical trials
Embryonic stem cells	Effectively differentiate into all three primary germ layers	Ethical/political issues surrounding the use of these cells	ESCORT
iPSs	Demonstrate an embryonic-like state and can be derived from somatic cells Strong regenerative capacity and integration within host cardiomyocytes	Risk of teratoma formation, thus there is a need to direct differentiation before transplantation	None
MSCs	Immunomodulatory characteristics permits the potential to use allogeneic cells Self-renewal, proliferation and differentiation properties	More studies needed to support the efficacy of these cells on a long-term basis	BOOST, REPAIR-AMI,
Bone marrow	Immunomodulatory characteristics Easy to isolate via liposuction Rich source of stem cells		MySTromalCell Trial The Precise Trial
Adipose tissue			
CSCs	Express cardiac specific markers and can thus differentiate more effectively into cardiomyocytes compared to other cell types	Difficult to isolate/extract cells Need for ex vivo expansion before transplantation, which can be costly	SCIPIO, CADUCEUS

*iPSs* induced pluripotent stem cells; *MSCs* mesenchymal stem cells; *CSCs* cardiac stem cells

## Results

### Embryonic/induced pluripotent stem cells

Embryonic stem cells can give rise to all cell types found in an organism. They are derived from the inner cell mass of the blastocyst during mammalian embryonic development [5]. However, there are several ethical and political issues surrounding the use of embryonic stem cells as well as a limited supply of donor human embryos. This led to the development of induced pluripotent stem cells (iPSs). IPss were first reprogrammed from adult mouse fibroblasts by the presence of certain factors, including Oct ¾, Sox2, c-Myc and Klf4 in culture. These iPSs successfully demonstrated an embryonic-like state, exhibiting growth properties and specific marker genes of embryonic cells [6, 7]. Shortly later, human iPSs, capable of differentiating into cell types from all three germ layers, were reprogrammed from human fibroblasts using the same four factors [8, 9]. Research has continued to focus on optimizing techniques used to form human iPSs such as using different factors and culture conditions. For example, human iPSs were generated using Oct4, Sox2, Nanog and Lin28 transgenes [10, 11].

For clinical application, these human induced pluripotent stem cells (hiPSs) must be able to differentiate into functional cardiomyocytes or cardiac progenitors. There is a large body of research regarding various differentiation techniques. One research group made use of the transcription factors used to induce pluripotency to also encourage differentiation into cardiac lineages. Oct ¾ is a transcription organizer that plays a gatekeeper role in the pluripotency of embryonic stem cells by interacting with the Sox2 promoter. Once this is achieved, Oct ¾ interacts with the Sox17 promoter to signal cardiogenesis [12]. Another research group used the addition of particular growth factors, including BMP and GSK3, to direct differentiation into cardiac progenitor cells [13]. Cardiac differentiation was confirmed using an embryoid body protocol by electrophysiology studies that verified the formation of nodal, atrial and ventricular-type cardiac cells from these cells [10]. These studies confirm the ability for iPSs to differentiate into cardiac lineages, thus making them good candidates for the treatment of cardiovascular disease.

In order to evaluate the effect of these cells on heart function, differentiated cardiac cells then must be transplanted into hearts. Injection of iPSs into rat myocardium resulted in successful engraftment and differentiation into cardiomyocytes, vascular endothelium and smooth muscle cells along with an increased ejection fraction and decreased fibrosis [14, 15]. In a porcine model, hiPSC-derived cardiomyocytes, endothelial cells and smooth muscle cells were integrated into host myocardium using an intramyocardial microsphere transplantation technique. This resulted in improved LV function, myocardial metabolism and reduced infarct size [16]. In a larger animal model, cardiac committed ESCs were transplanted into infarcted sheep hearts. Results demonstrated successful engraftment and preservation of ejection fraction [17]. Overall, these in vivo studies demonstrate a positive effect of implanted cells on cardiac function.

Although studies mentioned above demonstrate successful use and effect on cardiac function of iPSs in animal studies, progress is still needed in clinical trials. Clinical trials have not yet been initiated for iPSs use for the treatment of myocardial infarction. Before this can be done, there is a need to establish a reproducible and more standardized differentiation technique. In terms of embryonic stem cells, the first clinical trial was recently initiated in Paris. A phase 1 clinical trial, ESCORT, is currently examining the effect of embryonic stem cell derived progenitors on severe heart failure. These cells are driven towards a cardiac fate before transplantation and are embedded into a fibrin gel before being administered via epicardial delivery to patients undergoing coronary artery bypass grafting (CABG) or a mitral valve procedure. The first clinical case report using embryonic stem cell derived cardiac progenitor cells was recently published and demonstrated symptomatic improvement and echocardiographically evident new contractility with an LVEF improving from 26 % to 36 % after 3 months [18].

Induced pluripotent stem cells are attractive because of their ability to differentiate into large numbers of cardiomyocytes. There is better functional integration within host heart cells in comparison to adult cells, specifically in terms of the electromechanical connections with host cardiomyocytes. A limitation to the use of these cells is the associated risk of teratoma formation after transplantation of undifferentiated cells into infarcted hearts. In one study, the transplantation of undifferentiated syngenic iPSs into mice resulted in teratoma formation in 65 % of transplantation sites after 30 days [19, 20]. This result emphasizes the need for a method to control or direct differentiation of iPS towards cardiac progenitor cells before transplantation to avoid the formation of teratomas or alternate and undesired cell types [21]. Overall, additional animal studies are needed to ensure the safety and efficacy of these cells.

### Mesenchymal stem cells

Mesenchymal stem cells (MSCs) can be found in bone marrow, adipose tissue, umbilical cord blood and placenta. They are capable of differentiating into lineages such as osteocytes, chondrocytes, adipocytes, myocytes and marrow stroma [22]. These cells are hypothesized to secrete soluble growth factors and cytokines that act in endocrine and paracrine fashions, contributing to their therapeutic effect [23].

**Fig. 1:**
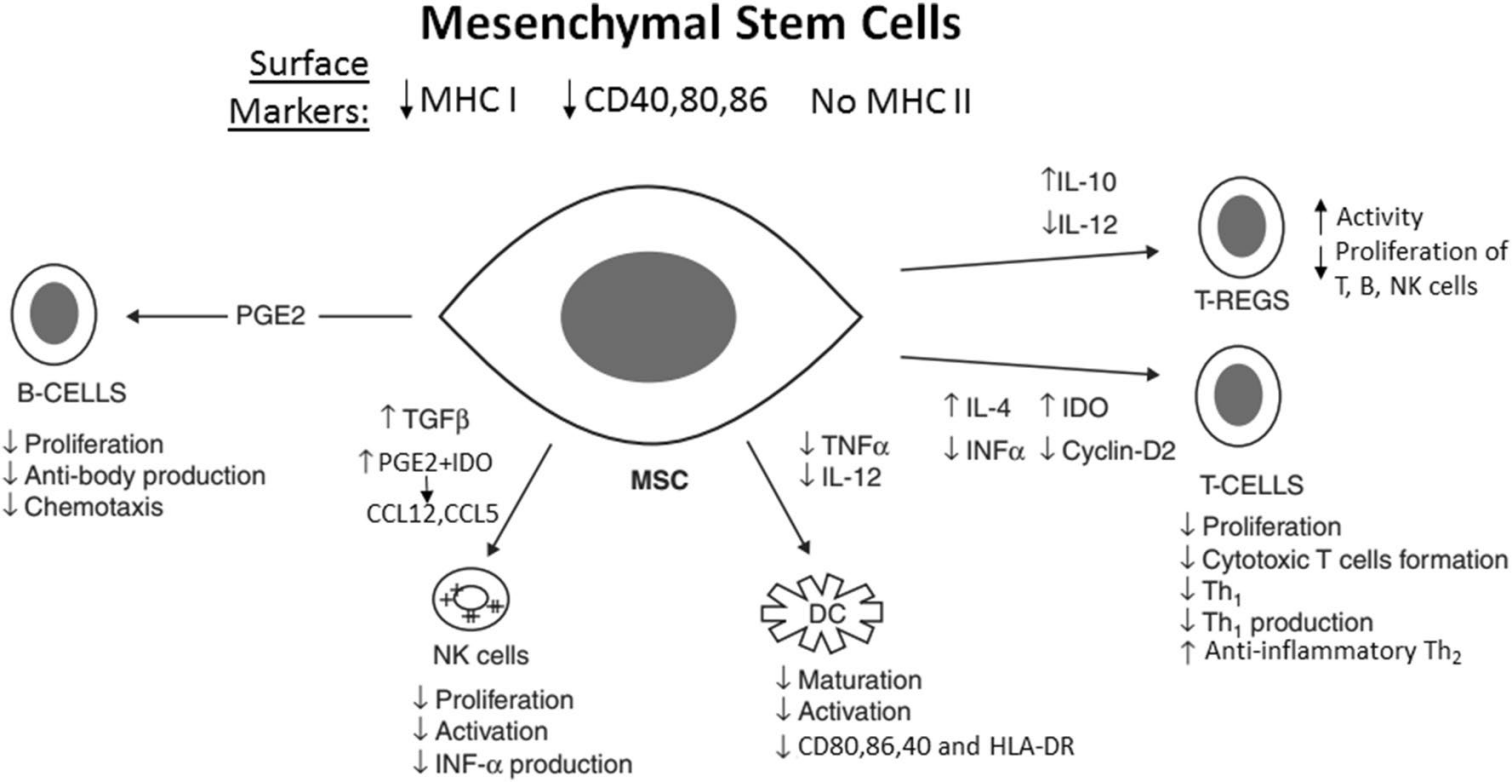
Suggested pathways underlying the immunomodulatory properties of mesenchymal stem cells This figure is adapted from [24]

MSCs are advantageous due to their immunomodulatory characteristics, which allow them to act as a universal reserve of donor cells (Fig. 1) [24]. These cells have a unique distribution of surface markers that allows them to escape detection from immune cells. Specifically, they possess decreased levels of MHC class I and costimulatory CD40, CD80 and CD86, with no MHC class II molecules [25, 26].

Despite low cellular retention and poor differentiation into cardiomyocytes, studies were still demonstrating improved heart function [27], which suggests a potential paracrine mechanism of action for transplanted MSCs. MSCs release angiogenic, apoptotic, mitogenic and homing factors that have cardio-protective actions [28]. Specifically, a recent study demonstrated the importance of hepatocyte growth factor (HGF) in angiogenesis and proliferation of cardiomyocytes [29]. Furthermore, transplantation of MSCs indirectly stimulated endogenous cardiac progenitor cells through a paracrine mechanism. Released factors such as HGF, VEGF and IGF-1 triggered activation, proliferation, and migration of resident cardiac progenitors. Specifically, they inhibited apoptosis of cardiac progenitors and upregulated the expression of cardiomyocyte related genes in order to direct differentiation [30].

Results from studies using MSCs derived from bone marrow and adipose tissue will be discussed below.

#### Bone marrow

Injury to an organ triggers stem cells from distant sites to migrate to the area of damage, where they can differentiate and help repair the damaged organ. This reparative effect had been demonstrated in muscle, liver and brain tissues [31–33]. In 2001, Orlic et al. investigated whether stem cells from a distant site directly transplanted into the heart could regenerate scarred areas. Results demonstrated that locally delivered autologous bone marrow stem cells could regenerate damaged myocardium in infarcted mice hearts [34]. This finding led to further research examining the differentiation of MSCs into cardiac lineages and the specific effects on cardiac function.

For example, in vitro MSCs were capable of differentiating into cardiomyocytes, as demonstrated when MSCs were co-cultured with neonatal ventricular myocytes [35]. In animal models, MSCs derived from bone marrow injected into mice and rat hearts post MI showed successful engraftment and differentiation into cardiomyogenic and vascular phenotypes [36–38]. It has also been found that the hypoxic environment of infarction may induce the expression of certain factors (vascular endothelial growth factor, myocardin, insulin like growth factor) which can promote the differentiation of MSCs to cardiomyocytes [39, 40]. Percutaneous transendocardial injection of bone marrow derived MSCs into pig hearts post-MI showed an improved ejection fraction after 4 weeks, in comparison to bone marrow derived mononuclear cells [41].

The above studies all used MSCs derived from autologous sources. However, as mentioned, an advantage of MSC is their immunomodulatory characteristics. This led to numerous studies comparing results from allogeneic vs autologous cells. *In vitro*, MSCs co-cultured with immune cells upregulated the secretion of suppressive cytokines (i.e. IL-4 and IL-10), decreasing the secretion of proinflammatory cytokines (TNF-α and IFN-ɣ) from dendritic cells, T helper cells and macrophages [42]. Soluble mediators including prostaglandin E2 are important for this immunosuppression [43]. In vivo, direct intramyocardial injection of bone marrow derived MSCs 1 month after MI in a porcine model resulted in preserved left ventricular ejection fraction at 60 and 90 days post MI in comparison with the untreated subjects [44]. Similarly, global left ventricular ejection fraction (LVEF) improved along with a decrease in infarct size by 40 % in sheep treated with intracoronary infusion of allogeneic bone marrow derived MSCs in comparison to untreated sheep [45]. With this being said, there is a lack of evidence supporting the benefit of these cells on a long-term basis. For example, intramyocardial injection of MSCs resulted in an initial improvement in cardiac function. This effect was no longer observed after 1 month, and there was noted immune cell infiltration and subsequent rejection of MSCs [46]. Preliminary results demonstrate benefit from the use of allogeneic MSCs. Future studies that focus on enhancing the long term efficacy of these treatments could permit the use of cells from young healthy donors, eliminating the need for MHC matching prior to treatment.

There have been numerous clinical trials evaluating the effect of MSCs in humans with cardiovascular disease. The BOOST trial involved intracoronary administration of autologous bone marrow derived MSCs in patients who received percutaneous coronary intervention for acute STEMI’s. After 6 months, the global LVEF increased by 6.7 % in the treated group vs 0.7 % in the control group [47]. Another trial in 2006 assessed cardiac function after intracoronary administration of autologous bone marrow derived MSCs 3–7 days post-MI. After 4 months the LVEF increased by 5.5 % in the treated group vs 3 % in the control group [48]. A phase I trial initiated in 2009 evaluated the safety and efficacy of bone marrow derived allogeneic MSCs (Prochymal) delivered intravenously to patients post-MI. Results demonstrated better global symptom scores and ejection fractions in patients receiving MSC’s compared to the control group. Improvements in LVEF were also seen on cardiac MRI in the group treated with MSCs [49].

#### Adipose tissue

MSCs can also be isolated from adipose tissue, specifically the stromal fraction of adipose tissue. These cells are attractive due to the ease of extraction using liposuction. Adipose tissue is also considered a richer source of progenitors compared to bone marrow, containing 100–500 times the amount [50].

In animal models, adipose tissue derived MSCs injected into rat myocardium post-MI resulted in an improved LVEF compared to untreated rats [51], as well as reduced fibrosis and less wall thinning [52]. More recently, adipose tissue derived stem cells were used to create cardiac-like progenitors (iCPs). Adipose tissue derived stem cells, induced cardiac progenitors and bone marrow derived stem cells were delivered into mice hearts post-MI. At 1 month post-transplant, mice transplanted with iCPs and adipose derived stem cells showed higher myocardial capillary densities. All three samples showed a decrease in infarct size compared to the control or untreated sample; however the greatest reduction was seen in those transplanted with iCPs, which were derived from adipose tissue stem cells [53].

Similar to bone marrow MSCs, those derived from adipose tissue also possess immunomodulatory properties. In vitro, MSCs derived from adipose tissue showed greater immunosuppressive effects when cultured with immune cells than MSCs derived from bone marrow or umbilical cord matrix. Specifically, they showed a greater inhibitory effect on CD4+ and CD8+ T cell activation and natural killer cell activation. They also displayed a suppressive effect on B cells [54]. In a separate study, adipose tissue MSCs were found to inhibit B cell function more effectively than bone marrow derived cells [55]. These studies indicate the effective immunosuppressive characteristics of MSCs derived from adipose tissue, supporting their use as a future therapeutic agent.

The mesenchymal stromal cell therapy in patients with chronic myocardial ischemia (MyStromalCell Trial) is a phase II study using adipose derived cells, stimulated with VEGF-A_165_, to determine the effect on patients with chronic ischemic heart disease and refractory angina [56]. The Precise Trial is using adipose tissue derived cells for transendocardial injections in patients with ischemic cardiomyopathy. Preliminary results show improvements in left ventricular mass and motion score index in treated patients after 18 months [57].

These cells continue to be attractive due to their ease of isolation via liposuction and their immunosuppressive qualities.

### Cardiac stem cells

Until recently, it was believed that myocytes were incapable of regeneration; however, evidence suggests that myocytes can mitotically divide post MI [58]. The same author studied a subpopulation of cells found within the heart, and proved that they hold properties of cardiac stem cells, with the potential to give rise to myocytes, smooth muscle and endothelial cells [59]. These cells were found to express markers of both mesenchymal stem cells (CD90, CD105), embryonic stem cells (Rex1, Nanog, Sox2) and also early markers of cardiogenesis (platelet derived growth factor receptor-α) [60, 61]. There are multiple different types of cardiac stem cells (CSCs) including ckit+ cells, Isl 1+ cells, cardiac mesoangioblasts, cardiosphere derived cells and epicardial progenitors which all express slightly different but overlapping surface markers [3].

Cardiac stromal cells can be isolated from adult human auricles. These cells expressed cardiovascular markers more efficiently than bone marrow MSCs in vitro. In vivo, cells originating from cardiac tissue differentiated more effectively into cardiomyocytes when injected into rat myocardium post MI [62]. Another study compared the effects of adipose tissue derived stem cells from subcutaneous vs pericardial origin. The cells from a pericardial origin showed better expression of intrinsic transcription factors for cardiogenesis. After administration of cells to infarcted hearts, they demonstrated increased vasculogenesis and myogenesis as well as more effective overall reparative activity [63]. Animal studies consistently demonstrate that cardiac stem cells possess a greater ability to differentiate into cells of cardiac lineages and can more efficiently obtain structural characteristics of myocytes and vessels [64]. Therefore, they are an attractive option for future therapeutics for cardiovascular disease.

Several studies have focused on the use of cardiosphere derived stem cells for treatment post-MI. To generate these cells, percutaneous endomyocardial biopsied cells are grown in culture to form cardiospheres which are then expanded ex vivo to generate cardiosphere derived cells before transplantation. Human and porcine cardiosphere derived cells successfully expressed antigenic characteristics of stem cells. In animal studies, injections of human derived cells into infarcted myocardium resulted in improved LVEF in mice [65] and the formation of new cardiac tissue and a reduction in infarct size in porcine [66]. A challenge associated with the use of cardiac stem cells is harvesting technique. In most studies, autologous cardiac stem cells are isolated from atrial appendages, ventricles or epicardial biopsies and are expanded in culture before use [67]. This method is advantageous because it provides a feasible technique to generate a larger number of cardiac stem cells from very small biopsies.

Clinical trials have already begun. The stem cell infusion in patients with ischemic cardiomyopathy (SCIPIO) is a phase I clinical trial administering autologous cardiac stem cells to patients with heart failure undergoing CABG. Preliminary results demonstrate an increase in LVEF at 4 and 12 months after infusion as well as a decrease in infarct size [68]. The Intracoronary cardiosphere-derived cells for heart regeneration after myocardial infarction (CADUCEUS) trial is a phase 1 study examining the use of autologous cells grown from percutaneous endomyocardial biopsies. MRI results after 6 months showed reductions in scar mass, increases in viable heart mass and contractility in the treated group [69].

An obstacle to the use of cardiac stem cells would be the fact that they need to be expanded ex vivo before transplantation, which can be costly [70]. Non-specific differentiation into other lineages such as adipocytes and skeletal muscle has been observed on occasion [71, 72]. Some techniques are focusing on activating endogenous cardiac stem cell pools using growth factors, microRNAs or drugs rather than actually transplanting cells. For example, one study identified a hepatocyte growth factor and insulin growth like factor receptor system in cardiac stem cells. Infarcted dog hearts were injected with these factors, resulting in the formation of new myocytes and coronary vessels in the infarcted region, with expressed proteins specific for cardiomyocytes [73]. This method would avoid complications associated with ex vivo expansion of cells.

Despite the challenges, studies have consistently demonstrated that cardiac stem cells possess the ability to more efficiently differentiate into cardiomyocytes and vascular endothelial cells as well as improve cardiac function [74]. There is also potential for expansion to treat a variety of cardiovascular diseases. For example, a study in 2014 even demonstrated the capability of CSCs isolated from mouse hearts to differentiate into sinus node like cells when co-cultured with mouse sinus node tissue [75]. This would have potential clinical applications for the treatment of sick sinus syndrome. More studies are needed to determine the feasibility of using cardiac stem cells.

## Conclusion

Myocardial infarction is a leading cause of death worldwide. The use of stem cells to regenerate damaged myocardium and restore function continues to be a potential therapeutic option. Several different types of stem cells have been investigated for this purpose. Ideal stem cells must be capable of differentiating into cardiomyocytes and vascular endothelial cells. They must be able to successfully engraft and integrate within the host myocardium, mechanically and electrically [67] or provide benefit via paracrine mechanisms [30]. It is important that the source of cells demonstrates cardiogenic potential. Another consideration is the ease of isolation.

There are ethical issues regarding the use of embryonic stem cells. However, induced pluripotent stem cells formed from fully differentiated somatic cells were found to possess an embryonic-like state. These cells have the ability to easily differentiate into large numbers of cardiomyocytes [14]. However, the use of proliferative undifferentiated pluripotent cells carries the risk of teratoma formation. Therefore, it is essential to differentiate the cells into cardiac lineages prior to transplantation.

Mesenchymal stem cells can be isolated from several sources including bone marrow and adipose tissue. Both sources have demonstrated successful differentiation into cardiac lineages as well as positive impacts on cardiac function in animal models. An advantage of mesenchymal stem cells is their immunomodulatory properties. It has been shown that there is a limited capacity for proliferation in cells from aged individuals who may or may not have additional comorbidities [76]. This would permit the use of allogeneic stem cells from young healthy donors for elderly patients to treat acute MI.

Studies have consistently demonstrated that cardiac stem cells possess the ability to more efficiently differentiate into cardiomyocytes and vascular endothelial cells as well as improve cardiac function. However, these cells may be more difficult to obtain compared to the other cell types. In one study, cardiac stem cells were isolated via endomyocardial biopsies of explanted hearts or at the time of left ventricular assist device implantation. This research showed that growth characteristics of cells taken from small endomyocardial biopsies (5 mg in size) in patients with heart failure were comparable to those from larger biopsies [77]. Although these samples are more difficult to obtain, studies are focusing on the use of expansion ex vivo before transplantation into damaged hearts.

Overall, this research offers a new method to treat myocardial infarction. Each cell type has its own benefits and limitations but more studies that directly compare the effectiveness of each cell type are warranted. Initially, studies focused on regeneration of myocardium for ischemic heart disease; however, studies are now expanding to include a larger spectrum of cardiovascular diseases.

## Abbreviations

MI: myocardial infarction
iPSs: induced pluripotent stem cells
hiPSs: human induced pluripotent stem cells
MSCs: mesenchymal stem cells
LVEF: left ventricular ejection fraction
iCPs: cardiac like progenitors
CSCs: cardiac stem cells
CABG: coronary artery bypass grafting
